# Medicine affordability and access in India: lessons from generic–branded price variation under the Jan Aushadhi Scheme

**DOI:** 10.3389/fpubh.2025.1629835

**Published:** 2025-12-05

**Authors:** Deepak Kumar Behera, Dil B. Rahut, Mehnaaz Dhanal Mehboobali, Shaik Husna Tasneem, Ambigai Rajendhran

**Affiliations:** 1 Economics and Finance Department, The Business School, RMIT International University Vietnam, Ho Chi Minh City, Vietnam; 2 Vice Chair and Senior Research Economist, Asian Development Bank Institute (ADBI), Tokyo, Japan; 3 Research Associate, College of Medicine and Public Health, Flinders Health and Medical Research Institute, Flinders University, Bedford Park, SA, Australia; 4 Research Associate, EVA Cytel, Hyderabad, India; 5 Manipal School of Commerce and Economics, Manipal Academy of Higher Education (MAHE), Manipal, Karnataka, India

**Keywords:** medicine affordability, drug price variation, generic medicines, branded medicines, Jan Aushadhi Scheme (JAS), out-of-pocket expenditure

## Abstract

**Background:**

High out-of-pocket expenditure (OOPE) on medicines continues to be a major driver of health-related financial hardship in India. The Jan Aushadhi Scheme (JAS), launched by the Government of India, seeks to improve affordability and access to essential medicines through generic substitution. However, systematic evidence on its pricing dynamics, affordability, and equity implications remains limited.

**Methods:**

We conducted an observational analysis of 696 branded formulations for Eye and Ear, Nose, and Throat (ENT) conditions, comparing their prices with Jan Aushadhi generics listed in the Pharmaceuticals and Medical Devices Bureau of India (PMBI). Data were drawn from the Current Index of Medical Specialties (CIMS, 2021) and PMBI catalogs. Cost ratios and price variation percentages were calculated, alongside affordability assessment using the WHO/HAI one-day wage benchmark. Correlation and regression models assessed the relationship between brand proliferation and price dispersion. Supplementary analyses included chronic disease medicines (diabetes, hypertension, and insulin), distribution trends of Jan Aushadhi Kendras (2018–2022), and global benchmarking using WHO indicators.

**Results:**

Branded formulations exhibited wide cost variation, with correlation analysis showing that greater brand numbers were associated with higher—not lower—price dispersion. While most JAS medicines met the WHO affordability threshold, chronic therapies such as glaucoma drugs remained financially burdensome for low-income households. Supplementary analyses demonstrated that dosage form, packaging, and storage requirements contribute to persistent price gaps. Geographic analysis revealed strong growth in Kendras (3,200 in 2018 to 9,000 in 2022), but significant inequities persisted, with large states such as Uttar Pradesh and Bihar showing low per-capita availability. Global benchmarking highlighted India’s paradox: despite being a global leader in generic production, domestic uptake of generics remains far below comparator countries.

**Conclusion:**

The Jan Aushadhi Scheme has made measurable progress in improving medicine affordability, but inequities in access, persistent price variation, and limited uptake constrain its full potential. Strengthening regulatory oversight, improving geographic distribution, and addressing physician and patient perceptions of generics are essential to maximize policy impact. Future research should apply quasi-experimental methods and integrate patient perception data to better capture the affordability–access–adherence pathway.

## 1 Introduction

Modern medicine is globally recognized for its transformative role in managing disease and improving population health (1, 2). However, rising pharmaceutical costs continue to challenge both health systems and households, particularly in low- and middle-income countries. In India, this issue is particularly acute. Out-of-pocket expenditure (OOPE) remains the dominant mode of financing healthcare, accounting for nearly 40% of total health spending, among the highest levels worldwide (3). Medicines constitute the largest share of this OOPE burden, representing more than half of household healthcare spending in some states (3, 4).

The implications of this financial pressure are far-reaching. Poor and middle-income families often spend a substantial portion of their earnings on medicines, with catastrophic expenditure leading to indebtedness and impoverishment (3). For chronic non-communicable diseases such as diabetes, hypertension, and cardiovascular disorders, the financial burden is even greater due to the lifelong nature of treatment (4). This entrenched reliance on out-of-pocket spending underscores the urgency of developing sustainable policy interventions that can reduce costs and improve equitable access to essential medicines.

In response to these challenges, the Government of India launched the Jan Aushadhi Scheme (JAS) in 2008. The initiative aimed to promote access to affordable, quality-assured generic medicines through a dedicated network of Jan Aushadhi Kendras.^1^ Initially, the scheme expanded slowly, with fewer than 100 Kendras operational by 2015 (5). The program was significantly restructured in 2016 as the Pradhan Mantri Bhartiya Jan Aushadhi Pariyojana (PMBJP), which broadened its scope and introduced stronger quality assurance systems. Since then, the scheme has grown rapidly: from roughly 3,200 Kendras in 2018 to more than 9,000 by 2023, with an official target of 10,500 Kendras by 2025 (6). Alongside the network’s expansion, sales have increased substantially, reaching more than ₹890 crore in 2022 (6). Under PMBJP, the product portfolio has expanded to over 1,450 medicines and 200 surgical items. All generics are procured from WHO-GMP certified manufacturers, and each batch undergoes quality testing in NABL-accredited laboratories before distribution (6). These mechanisms aim to build public confidence and ensure that generic medicines meet global quality standards.

Despite this progress, however, utilization of Jan Aushadhi outlets remains uneven, with strong uptake in some regions and minimal penetration in others. Understanding the factors that drive or hinder adoption is therefore critical for maximizing the scheme’s impact. Generic medicines are therapeutically equivalent to branded formulations in terms of active ingredients, safety, dosage, and efficacy (7, 8). Economically, they represent a cost-saving alternative by reducing expenditure on research, marketing, and brand promotion. Yet despite these advantages, adoption of generics in India has lagged behind expectations.

A persistent challenge is physician and pharmacist bias. Studies have shown that prescribers often favor branded drugs, influenced by perceptions of superior quality or by direct and indirect marketing from pharmaceutical companies (9–11). Patients, too, may distrust generic medicines, associating lower cost with inferior quality (12, 13). This “perception barrier” is compounded by the confusing proliferation of brand names in India’s pharmaceutical market (10), making it difficult for patients to distinguish between genuine generics, branded generics, and originator drugs. Efforts to strengthen confidence in generics have included awareness campaigns, prescribing guidelines, and quality audits. Nevertheless, the entrenched dominance of branded prescribing practices remains a formidable obstacle. Addressing these biases is essential for ensuring that Jan Aushadhi medicines reach the populations most in need.

India’s position in the global pharmaceutical landscape highlights a striking paradox. The country is the world’s largest producer and exporter of generic medicines, supplying affordable drugs to markets across Africa, Latin America, and Asia (14). Yet domestically, the penetration of generics remains exceptionally low. Recent data from the World Health Organization’s Global Health Observatory (2023) reveal that the median availability of generics in India is only 1.4%, compared to much higher levels in countries such as Bangladesh, Sri Lanka, Brazil, and South Africa, where availability ranges between 20 and 45% (15, 16). Moreover, the median price ratio of generics to branded medicines in India is nearly 60%, compared with ratios of just 8–18% in comparator countries (15).

This paradox underscores systemic weaknesses in India’s internal pharmaceutical ecosystem. Despite its role as a global hub for generic production, the domestic market continues to rely heavily on branded medicines. This disconnect reflects not only supply-side issues (distribution, procurement, and supply chain constraints) but also demand-side challenges (prescriber preferences and patient perceptions). It highlights the urgent need for stronger domestic policy mechanisms to translate India’s manufacturing capacity into equitable access for its own population.

While previous research has examined the affordability of medicines in India, significant knowledge gaps remain. Most studies have focused on broad comparisons between generics and branded drugs (17, 18), with limited attention to therapeutic areas such as Ear, Nose, and Throat (ENT), which represent a substantial share of outpatient morbidity and disability (19, 20). Moreover, few studies have systematically analyzed cost variation within the branded segment, or identified lower-cost branded alternatives that could offer immediate savings for patients.

This study addresses these gaps by providing: (1) A systematic price comparison between Jan Aushadhi generics and branded medicines in the Indian market. (2) Identification of less-expensive branded alternatives, offering new insights into intra-brand price variation. (3) A specialized focus on ENT medicines, which remain underexplored in affordability research despite their significant disease burden. (4) Integration of updated global evidence to situate India within broader international patterns of generic availability and pricing. (5) An evaluation of PMBJP’s expansion and prospects, linking affordability analysis with policy implications for OOPE reduction and equity in access.

By combining national and international perspectives, the study provides a comprehensive and policy-relevant assessment of medicine affordability in India. Its findings aim to inform policymakers, healthcare practitioners, and civil society actors seeking to improve the reach and impact of generic medicines in reducing OOPE and enhancing equitable healthcare access.

## 2 Methods

### 2.1 Study design and scope

This study employs an observational and analytical design to compare the prices of medicines listed under the Jan Aushadhi Scheme (JAS) with equivalent branded formulations used for Eye and Ear, Nose, and Throat (ENT) conditions. The dataset comprised 696 branded medicines, including both single and combination formulations manufactured by different companies across these therapeutic areas.

The decision to focus on Eye and ENT medicines was informed by their public health relevance. According to Global Burden of Disease (GBD) estimates, hearing impairment is among the top three causes of disability worldwide, while uncorrected vision impairment and cataracts remain leading contributors to avoidable blindness. Despite this burden, affordability assessments of these therapeutic categories in India remain scarce, which justifies their selection as the core scope of the present analysis.

### 2.2 Data sources

Data on branded drug prices were obtained from the *Current Index of Medical Specialties (CIMS)* for the period September–November 2021, while prices of generics supplied under JAS were sourced from the Pharmaceuticals & Medical Devices Bureau of India (PMBI) (25, 26). Both datasets report prices in Indian Rupees (INR). Medicines absent from either source were excluded.

Additional datasets were used to strengthen the scope of the analysis: Cross-country data on generic availability and pricing were drawn from the World Health Organization (WHO) Global Health Observatory (15). Information on the expansion of Jan Aushadhi Kendras (2018–2022) was obtained from official *PMBJP reports*. State-level distribution of Kendras was compiled from PMBI sources.

### 2.3 Analytical framework

(1) Cost ratio and variation analysis

The *Cost Ratio (CR)* was defined as the ratio of the highest-priced branded version of a formulation to the lowest-priced version. The *Cost Variation (CV)* was calculated as:

$\mathrm{CV}=\frac{\mathrm{HBP}-\mathrm{LBP}}{\mathrm{LBP}}\times 100$ .

where HBP = highest branded price and LBP = lowest branded price. These measures capture both the dispersion of branded prices and the relative affordability of JAS generics.

(2) Affordability assessment

Affordability was assessed using the WHO/HAI benchmark, expressed as the number of daily wages of the lowest-paid unskilled worker required to purchase a full treatment course.

(3) Correlation and regression analysis

Pearson’s correlation was used to test the relationship between the number of brands and cost variation. Multiple regression models were also estimated with cost variation as the dependent variable, and number of brands, formulation type (single vs. combination), and therapeutic category (Eye vs. ENT) as explanatory variables. This framework enabled testing whether greater brand competition reduces or amplifies price variation.

(4) Trend and distribution analysis

Time-series analysis was used to track the expansion of Jan Aushadhi Kendras between 2018 and 2022. State-level analysis identified the top five and bottom five states by Kendra distribution in 2022. To examine equity, population-adjusted ratios (Kendras per million population) were also calculated. Cross-country comparisons placed India’s generic availability and pricing alongside comparator countries using WHO datasets.

(5) Descriptive statistics and visualization

All data handling, descriptive statistics, and visualization were performed using Microsoft Excel (2019). Results are reported in tables, graphs, and geographic maps for clarity and comparability.

(6) Supplementary chronic disease analysis

To extend generalizability beyond Eye and ENT, three high-burden chronic medicines were included in supplementary analysis in Appendix Table A2: Metformin 500 mg, Amlodipine 5 mg, and Human Insulin 40 IU/mL. For each, branded prices (lowest, highest, and median) from CIMS were compared against JAS prices from PMBI for identical pack sizes. The cost ratio, cost variation, and savings versus the median branded price were calculated. Monthly affordability was then estimated under standard dosing assumptions (Metformin: 60 tablets/month; Amlodipine: 30 tablets/month; Insulin: 2 vials/month), expressed as days of wage using the WHO affordability parameter.

(7) Dosage form variation analysis

To capture the role of formulation differences in shaping price disparities, we compiled illustrative comparisons across dosage forms for selected Eye and ENT medicines (e.g., eye drops versus ointments). For each formulation, branded price ranges (highest, lowest, and median) were compared against JAS prices, and cost ratios and price variations were computed. This supplementary analysis in Appendix Table A3 highlights how packaging, storage, and dosage form contribute to price dispersion beyond brand competition.

### 2.4 Limitations

Several methodological limitations should be acknowledged. First, CIMS does not capture all marketed brands, while PMBI data are restricted to JAS-listed drugs, which may underestimate the full extent of market variation. Second, affordability estimates are based on national wage averages and do not capture regional disparities in income levels. Third, WHO cross-country datasets differ in survey years and data collection methods, which may affect comparability. Finally, while the study primarily focuses on Eye and ENT medicines, supplementary analysis of chronic drugs was limited to three illustrative examples and may not fully represent all therapeutic categories.

## 3 Results

### 3.1 A comparative analysis of generic medicine availability and pricing in India and global markets

**Figure 1:**
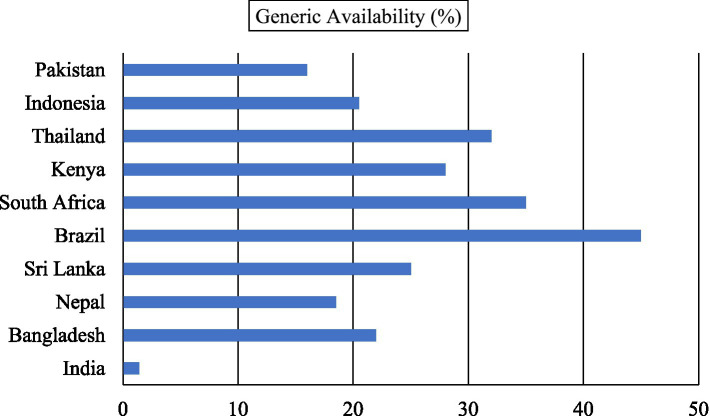
Median availability of selected generic medicines as a percentage of total medicines, 2018–2021. Source: Author(s) estimation from the data obtained from the Global Health Observatory, World Health Organisation (15).

**Figure 2:**
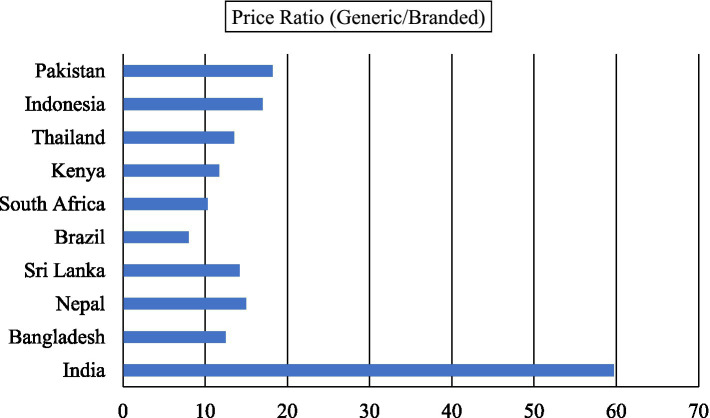
Median consumer price ratio of generic vs. branded, 2018–2021. Source: author(s) estimation from the data obtained from the Global Health Observatory, World Health Organisation (15).

Figures 1, 2 present an updated cross-country comparison of the availability and pricing dynamics of generic and branded medicines using data from 2018 to 2021. This evidence provides a clearer understanding of India’s relative position in the global pharmaceutical landscape.

Figure 1 shows a striking disparity in the availability of generic medicines across countries. India records a median availability of only 1.4 percent, far lower than its regional neighbors such as Bangladesh (22 percent), Nepal (18.5 percent), and Sri Lanka (25 percent). Emerging economies outside South Asia perform even better, with Brazil (45 percent), South Africa (35 percent), and Thailand (32 percent) reporting far higher levels of generic availability. These findings underscore a structural imbalance in India’s pharmaceutical market, where reliance on branded medicines continues to dominate despite policy initiatives to expand generics.

Figure 2 highlights the extent of price differentials between generics and branded medicines. India’s price ratio of 59.7 indicates that generics are comparatively more expensive relative to their branded counterparts when benchmarked against other countries. By contrast, countries such as Brazil (8.0), South Africa (10.3), and Bangladesh (12.5) demonstrate significantly lower ratios, reflecting more efficient adoption of generic alternatives. Even within South Asia, India lags, as Nepal (15.0) and Sri Lanka (14.2) show far narrower price gaps.

Taken together, the updated figures reveal that India continues to face a dual challenge of low availability and high relative prices for generic medicines. This reinforces the country’s heavy dependence on branded drugs and highlights the barriers that persist in fostering widespread adoption of affordable alternatives. Given the high burden of out-of-pocket expenditure on medicines in India, the Jan Aushadhi Scheme (JAS) assumes even greater importance. By improving both the supply and affordability of generics, JAS has the potential to reduce financial stress on households and to realign India’s pharmaceutical market with global best practices.

In summary, the expanded cross-country evidence demonstrates that India remains an outlier in generic medicine adoption, lagging well behind both regional neighbors and middle-income comparators. These insights strengthen the argument for strategic interventions, including regulatory reforms, prescriber sensitization, and scaling up of the Jan Aushadhi network, to ensure that generics fulfill their promise as a cost-effective healthcare solution.

### 3.2 Cost comparison of generic and branded medicines of EYE and ENT

An in-depth analysis was conducted on the prices of both generic and branded Eye medicines, encompassing a total of 26 generic pharmaceuticals and 559 branded Eye drugs, each with varying packaging units. Notably, the majority of the analyzed drugs were single entities (refer to Appendix Table A1). Cost variation was calculated using the formula suggested by literature (21). Among the Eye medications, specific drugs exhibited diverse patterns. For instance, Ofloxacin Eye Drops had the highest number of branded counterparts (131), followed by Tobramycin (80) and Timolol Maleate (73). In contrast, Olopatadine Hydrochloride had only one branded counterpart. Timolol Maleate 3 mL stood out with the highest cost ratio of 21.6, signifying its status as the most expensive drug in comparison to the least expensive drug, Olopatadine Hydrochloride. The range of cost variation spanned from 7.1 percent for Gentamicin 0.3 mL to a substantial 2055.9 percent for Timolol Maleate 3 mL.

### 3.3 Cost difference between JAS Price and branded minimum price

Simultaneously, the analysis extended to ENT medications, considering seven drugs from the Jan Aushadhi Scheme (JAS) drug list and their 137 branded counterparts. The analysis revealed intriguing findings. Paradichlorobenzene, Benzocaine, Chlorbutol, and Turpentine Oil at 10 mL demonstrated the highest price variation of 500 percent, while Fluticasone Propionate Nasal Spray exhibited the lowest variation at 8.5 percent. Paradichlorobenzene, Benzocaine, Chlorbutol, and Turpentine Oil at 10 mL also recorded the highest cost ratio at 6.0.

**Figure 3:**
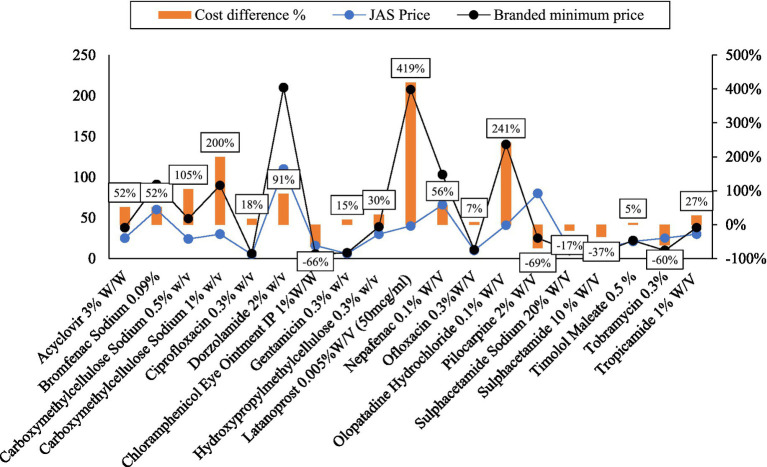
Percentage cost variation (“Cost difference %”) between minimum branded price and JAS price for eye drugs. Source: author’s estimation.

**Figure 4:**
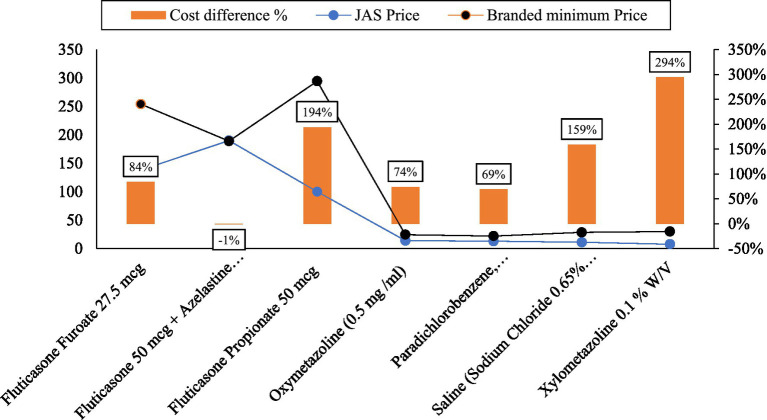
Percentage cost variation (“Cost difference %”) between minimum branded price and JAS price for ENT drugs. Source: author’s estimation.

Figures 3, 4 visually represent the cost variation between the minimum branded price and the Jan Aushadhi Scheme (JAS) price for Eye and ENT drugs, respectively. The comparative analysis indicates that not all generic drugs are uniformly cheaper than their branded counterparts. Specifically, the JAS price for eye medications was found to be 69 percent higher than branded drugs. Remarkably, substantial variations were observed, with the highest differences noted for Latanoprost (419 percent) and Xylometazoline (294 percent). Cipla emerged as the leading manufacturer, producing the highest number of Eye and ENT medications (28), followed by Intas (19), Allergan (18), and FDC (18). Notably, Timolol Maleate eye drops exhibited a notable cost variation, despite having a limited number of brands in the market. The correlation between cost variation percentage and the number of brands was moderately strong at 56 percent.

Table 1 provides a detailed cost comparison for selected ENT medicines, presenting information on JAS price, maximum and minimum prices per unit, average price per unit, cost ratio, and cost variation percentage.

**Table 1:** Cost comparison for ENT medicines.

Drugs and packaging unit	Therapeutic category	Count of brands	JAS price	Maximum per unit	Minimum price per unit	Average price per unit	Cost ratio	Cost variation %
Fluticasone 50 mcg + Azelastine 140 mcg Nasal	Corticosteroids	14						
70 MD		11	190	434.9	189.0	333.6	2.3	130.1
120 MD		3	NA	282.0	207.0	252.3	1.4	36.2
Fluticasone Furoate Nasal Spray 27.5 mcg	Corticosteroids	18						
70 MD		2	NA	434.9	396.0	415.5	1.1	9.8
100 MD		1	NA	350.0	350.0	350.0	1.0	0.0
120 MD		14	138	404.8	254.1	361.8	1.6	59.3
160 MD		1	NA	395.0	395.0	395.0	1.0	0.0
Fluticasone Propionate Nasal Spray 50 mcg	Corticosteroids	27						
70 MD		8	NA	385.0	304.4	341.9	1.3	26.5
100 MD		3	80	319.4	294.4	309.8	1.1	8.5
120 MD		16	NA	533.0	181.6	288.6	2.9	193.5
Oxymetazoline Nasal Drops (0.5 mg /ml)	Sympathomimetic agents	8						
10 ml		8	14	79.6	24.3	53.1	3.3	227.2
Paradichlorobenzene, Benzocaine, Chlorbutol and Turpentine Oil Ear Drops (2% + 2.7% + 5% + 15%)	Analgesic	25						
5 ml		1	NA	45.0	45.0	45.0	1.0	0.0
10 ml		24	13	132.0	22.0	57.4	6.0	500.0
Saline Nasal Drops (Sodium Chloride 0.65% w/v)	Sympathomimetic agents	29						
10 ml		18	NA	60.0	15.0	36.0	4.0	300.0
15 ml		9	NA	50.6	20.0	34.7	2.5	153.0
20 ml		2	11	42.3	28.5	35.4	1.5	48.4
Xylometazoline 0.1% W/V Nasal Drop	Sympathomimetic agents	16						
10 ml		16	7.6	50.6	30.0	40.1	1.7	69.0
Total		137						

Source: author’s estimation.

First: Fluticasone 50 mcg + Azelastine 140 mcg Nasal (Corticosteroids). The analysis covers 14 brands, primarily in 70 MD and 120 MD packaging units. JAS Price ranges from 189.0 to 434.9, with an average price of 333.6. The cost ratio indicates a variation of 2.3, suggesting some brands are 2.3 times more expensive than others. Cost variation is substantial at 130.1%, highlighting notable price diversity among brands.

Second: Fluticasone Furoate Nasal Spray 27.5 mcg (Corticosteroids). 18 brands are analyzed across different packaging units. JAS Price ranges from 350.0 to 434.9, with an average price of 415.5. Notably, one brand in 100 MD packaging has the same maximum, minimum, and average price, resulting in a cost ratio of 1.0 and zero cost variation. For the rest, the cost ratio varies, with a notable 59.3% cost variation.

Third: Fluticasone Propionate Nasal Spray 50 mcg (Corticosteroids). The analysis includes 27 brands with varying packaging units. JAS Price ranges from 304.4 to 533.0, averaging at 341.9. The cost ratio varies significantly, indicating substantial price diversity among brands. A notable 193.5% cost variation underscores the wide-ranging prices within this category.

Fourth: Oxymetazoline Nasal Drops (0.5 mg/mL; Sympathomimetic Agents). 8 brands are assessed in 10 mL packaging units. JAS Price ranges from 24.3 to 79.6, averaging at 53.1. The cost ratio is high at 3.3, reflecting considerable price differences. A significant cost variation of 227.2% highlights notable diversity in pricing.

Fifth: Paradichlorobenzene, Benzocaine, Chlorbutol, and Turpentine Oil Ear Drops (Analgesic). This category includes 25 brands primarily in 10 mL packaging units. JAS Price ranges from 22.0 to 132.0, with an average price of 57.4. The cost ratio is notably high at 6.0, indicating substantial price disparities. A substantial cost variation of 500.0% underscores the significant diversity in pricing.

Sixth: Saline Nasal Drops (Sodium Chloride 0.65% w/v; Sympathomimetic Agents). 29 brands are evaluated across 10 mL, 15 mL, and 20 mL packaging units. JAS Price ranges from 15.0 to 60.0, with an average price of 36.0. The cost ratio varies, indicating diverse pricing structures. A significant cost variation of 300.0% highlights the considerable price diversity in this category.

Seventh: Xylometazoline 0.1% W/V Nasal Drop (Sympathomimetic Agents). This category includes 16 brands in 10 mL packaging units. JAS Price ranges from 30.0 to 50.6, averaging at 40.1. The cost ratio indicates diverse pricing structures among brands. A notable cost variation of 69.0% underlines the variability in pricing within this category.

The analysis reveals substantial variations in pricing among different brands within each therapeutic category. The cost ratios and variation percentages highlight the complexity and diversity in the pricing of ENT medicines, emphasizing the need for strategies to promote affordability and accessibility, especially through initiatives like the Jan Aushadhi Scheme. In conclusion, the findings from this comprehensive analysis shed light on the intricate pricing dynamics of generic and branded medications for Eye and ENT conditions in India. The observed variations in cost, coupled with the impact of the Jan Aushadhi Scheme, underscore the complexity of pharmaceutical pricing and the potential avenues for enhancing affordability and accessibility.

### 3.4 Statistical analysis of cost variation and affordability

The combined statistical analysis provides further insights into the dynamics of drug pricing under the Jan Aushadhi Scheme. As shown in Table 2, the correlation between the number of available brands and the extent of cost variation was strong and positive (*r* = 0.79). This finding indicates that higher brand proliferation is associated with greater variation in pricing, contrary to the expectation that competition necessarily drives prices downward. Regression analysis reinforced this relationship, with the coefficient for brand count (15.86, *p* < 0.01) suggesting that, on average, each additional brand in the market increases the cost variation by approximately 16 percent (Table 3). The model explains around 62 percent of the variation (R^2^ = 0.62) in drug cost disparities, highlighting brand count as a significant driver of price differences in both Eye and ENT medicines. Affordability analysis (Table 4) based on the WHO/HAI method demonstrates that Jan Aushadhi medicines generally meet global benchmarks for affordability. For example, Ofloxacin eye drops (INR 10.7) and Xylometazoline nasal drops (INR 30.0) cost less than 0.1 days’ wage, reflecting high affordability. However, some medicines such as Latanoprost (0.55 days’ wage) and Fluticasone Propionate (0.81 days’ wage) approach or exceed half a day’s wage, suggesting a relatively higher burden for low-income households.

**Table 2:** Correlation and regression analysis of cost variation on brand count.

Test	Variable	Coefficient	Std. error	t-statistic	*p*-value	*R* ^2^
Correlation	Brands_Count vs. Cost_Variation	0.786	–	–	–	–
Regression	Constant	25.04	220.35	0.11	0.912	25.04
	Brands_Count	15.86	3.94	4.02	0.002	0.618

Source: author’s estimation.

**Table 3:** Affordability of JAS medicines (WHO/HAI method, daily wage = INR 375).

Medicine	Type	JAS price (INR)	Affordability index (days’ wage)
Timolol Maleate (Eye)	Eye	39.2	0.105
Ofloxacin (Eye)	Eye	10.7	0.029
Tobramycin (Eye)	Eye	10	0.027
Gentamicin (Eye)	Eye	6.9	0.018
Latanoprost (Eye)	Eye	207.6	0.554
Carboxymethylcellulose (Eye)	Eye	49	0.131
Fluticasone + Azelastine (ENT)	ENT	189	0.504
Fluticasone Propionate (ENT)	ENT	304.4	0.811
Oxymetazoline (ENT)	ENT	24.3	0.065
Paradichlorobenzene Combo (ENT)	ENT	22	0.059
Saline Nasal Drops (ENT)	ENT	15	0.04
Xylometazoline (ENT)	ENT	30	0.08

Source: author’s estimation.

**Table 4:** Top 5 and bottom 5 states by Jan Aushadhi Kendras (2022).

Rank	State	Kendras (2022)	Population (millions)*	Kendras per million population
1	Uttar Pradesh	1,250	230	5.4
2	Bihar	920	125	7.4
3	Maharashtra	870	125	7
4	Rajasthan	750	80	9.4
5	Madhya Pradesh	690	90	7.7
…	…	…	…	…
31	Goa	18	2	9
32	Nagaland	15	2.5	6
33	Sikkim	12	0.7	17.1
34	Mizoram	10	1.2	8.3
35	Andaman & Nicobar Is.	8	0.4	20

*Population estimates based on Census 2011 projections to 2022 (rounded).

To extend the analysis beyond Eye and ENT conditions, we examined three widely prescribed chronic disease medicines—Metformin, Amlodipine, and Human Insulin. The results (Appendix Table A2) reveal substantial brand-driven price variation, with cost ratios ranging from 3.2 (Insulin) to over 7.0 (Metformin). In all three cases, Jan Aushadhi formulations were consistently among the most affordable options. The savings were particularly notable for oral medicines such as Metformin and Amlodipine, where JAS prices were significantly lower than the branded median. However, for insulin, JAS formulations were cheaper than premium brands but not always the absolute lowest-cost option available. These findings demonstrate that patterns of wide price dispersion and affordability advantages for JAS drugs extend beyond the study’s core focus on Eye and ENT medicines, underscoring the broader policy relevance of the scheme for chronic disease management in India.

### 3.5 Distribution of Jan Aushadhi Kendras across Indian states and union territories

**Figure 5:**
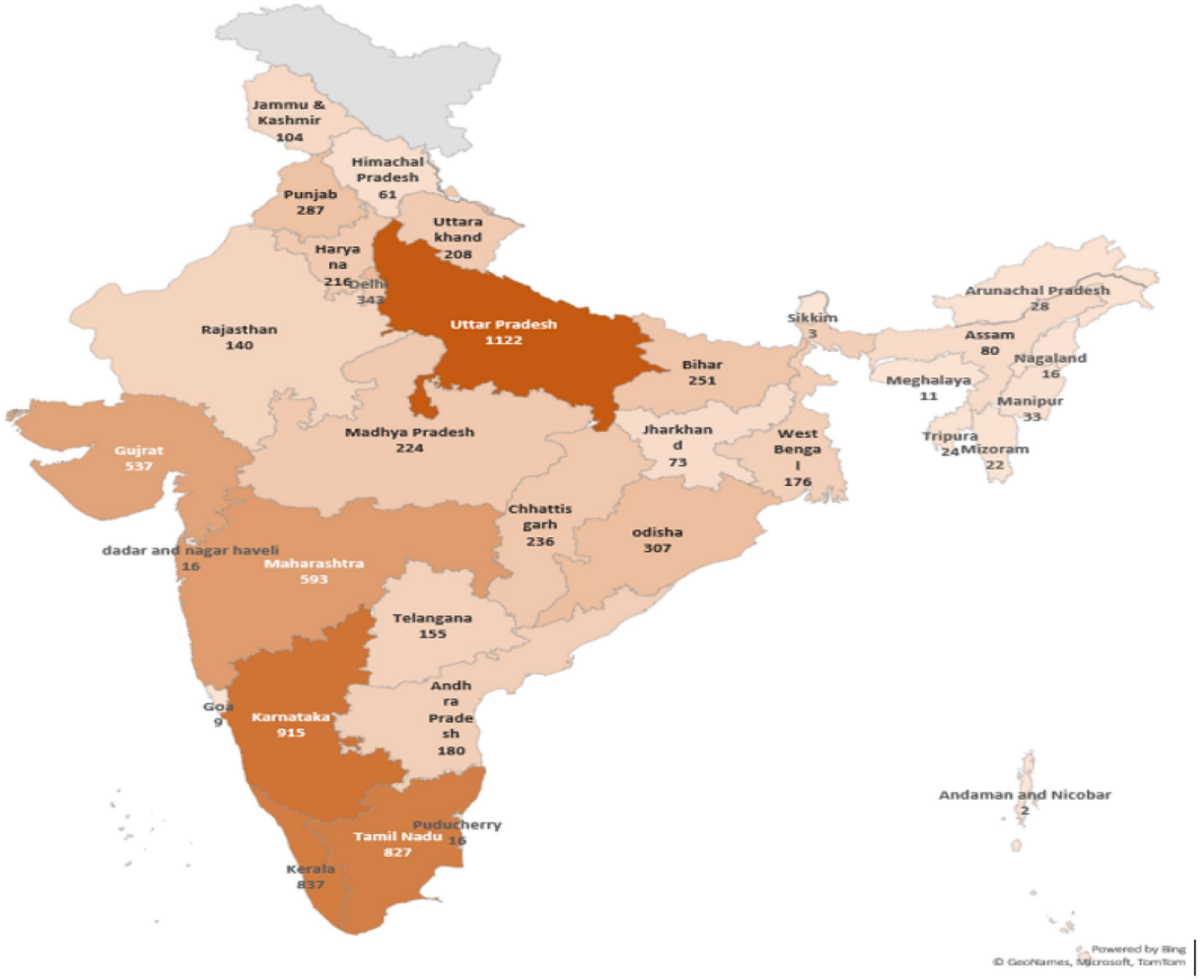
JAS Kendra’s across Indian States and Union Territories. Source: author’s estimation.

Figure 5 provides a visual representation of the distribution of Jan Aushadhi Kendras (JAS Kendras) across different Indian States and Union Territories. Geographical distribution is crucial in assessing the accessibility and availability of affordable generic medications provided by Jan Aushadhi. Several insights emerge from this analysis.

First, the figure highlights significant regional disparities. Uttar Pradesh stands out with the highest concentration, suggesting greater accessibility to Jan Aushadhi medicines, while Andaman and Nicobar Islands have only a handful of Kendras, indicating constrained access. Second, while concentration in populous states such as Uttar Pradesh and Bihar may suggest scale, the population-adjusted figures reveal much lower access than smaller states and union territories. Third, the overarching aim of Jan Aushadhi—providing cost-effective alternatives to branded medicines—is likely influenced by this uneven distribution, as equitable state-level coverage remains a challenge. Fourth, policymakers can use such geographic evidence to identify “high-demand, low-coverage” areas where future Kendras should be prioritized. Finally, awareness-building is particularly important in states with fewer Kendras to maximize uptake of available facilities.

#### 3.5.1 Trend analysis of Kendras (2018–2022)

**Figure 6:**
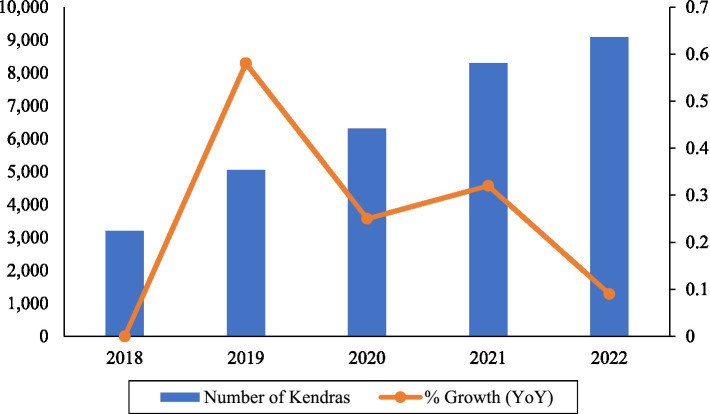
Expansion of Jan Aushadhi Kendras in India, 2018–2022. Source: author’s estimation based on PMBI reports.

Between 2018 and 2022, the number of Kendras nearly tripled, rising from 3,200 to over 9,000 (Figure 6). Growth was strongest between 2018 and 2019 (+58%), but has moderated in recent years, falling to just 9% in 2022. This pattern reflects an early acceleration in rollout, followed by stabilization as the scheme moved closer to saturation in some states. Nevertheless, the overall trajectory indicates that the government’s target of 10,500 Kendras by 2025 is within reach, provided the expansion pace is sustained.

#### 3.5.2 State-level distribution and equity

While the overall expansion is remarkable, distribution across states remains uneven. Table 4 presents both absolute numbers and population-adjusted access. States such as Uttar Pradesh and Bihar lead in absolute counts, but fall below 8 Kendras per million residents. In contrast, smaller states like Sikkim and union territories such as Andaman & Nicobar Islands achieve higher ratios despite low absolute numbers. This dual perspective underscores that equitable access remains a challenge, particularly in densely populated states.

## 4 Discussion

The results of this study contribute important evidence on the affordability, availability, and pricing dynamics of Jan Aushadhi Scheme (JAS) medicines in India, while also exposing the structural and behavioral challenges that limit their impact as suggested by previous literature (24, 27, 28). Our cost-variation and regression analysis show wide dispersion in branded drug prices for the same formulation, and the correlation results indicate that greater brand proliferation is associated with higher—not lower—price variability. This suggests that competition in India’s pharmaceutical market does not consistently deliver consumer savings; instead, brand proliferation often amplifies heterogeneity through mechanisms such as brand loyalty, marketing differentials, and segmented pricing strategies (21, 22, 23).

Supplementary evidence from chronic disease medicines (Appendix Table A2) demonstrates that similar dynamics extend beyond Eye and ENT drugs. Widely used treatments such as Metformin, Amlodipine, and Insulin show large branded price ranges and significant savings potential when substituted with JAS equivalents. This strengthens the generalizability of our findings, highlighting that the affordability challenges addressed here apply to chronic conditions as well, which account for the majority of household pharmaceutical spending in India.

The temporal and spatial analyses highlight strong but uneven progress in scheme expansion. The number of Kendras rose from approximately 3,200 in 2018 to over 9,000 in 2022, reflecting robust government commitment. However, population-adjusted comparisons reveal inequities: large states such as Uttar Pradesh and Bihar, despite hosting the highest absolute numbers of Kendras, register among the lowest ratios per million population. Conversely, small states and union territories with fewer absolute outlets often appear better served on a per-capita basis. Such disparities suggest that numerical growth alone may mask geographic shortfalls in access (29, 30, 31). Equity-oriented expansion, informed by population density and healthcare needs, will be critical to achieving the scheme’s poverty-reduction goals. Cross-country benchmarking further reveals that India lags comparator countries in generic penetration and generic-to-branded price ratios, despite its global role as a leading supplier of low-cost medicines (15, 32, 33, 34).

A key insight from the supplementary dosage-form analysis (Appendix Table A3) is that price variation is multidimensional. Differences in dosage form (e.g., drops versus ointments), packaging and storage requirements, cold-chain logistics, patent status, and procurement efficiency all contribute materially to cost disparities. For example, ophthalmic liquids attract consistently higher branded premiums, reflecting both handling costs and perceived clinical differentiation. These findings underscore that competition alone cannot ensure affordability, and that procurement reforms and formulation-specific cost controls are equally important.

Affordability benchmarking confirms that most JAS medicines fall within the WHO/HAI threshold of 1 day’s wage for the lowest-paid worker. However, affordability remains fragile for long-term treatments of chronic and ophthalmic conditions. For instance, while JAS prices reduce financial burden, even small recurring outlays accumulate into significant annual costs for poor households. In contrast, urban and wealthier patients often continue to purchase branded alternatives, influenced by prescriber behavior and perceptions of higher quality. This rural–urban divide underscores the importance of addressing both supply-side gaps (e.g., stock-outs, geographic maldistribution) and demand-side barriers (e.g., trust in generics, prescribing norms).

Illustrative cost-saving estimates highlight the scheme’s potential policy impact. For example, substituting branded Latanoprost with its JAS equivalent could save more than ₹5.5 crore annually per 1,000 patients requiring year-long treatment. While not causal evidence, such estimates demonstrate the scale of protection that wider generic adoption could deliver. Future research employing longitudinal and quasi-experimental approaches (e.g., difference-in-differences) would allow robust quantification of causal effects on market prices and household expenditures.

Patient perceptions remain a critical constraint on uptake. Evidence from NFHS-5 indicates that more than half of rural households cite cost and drug availability as barriers to healthcare access, and doubts about generic quality are consistently reported (11, 12, 35, 36, 37). Without improvements in public trust, affordability gains may remain underutilized. Integrating patient perception data into future evaluations will be essential to strengthen the affordability–perception–adherence framework.

Several limitations should be acknowledged. First, the analysis relies on 3 months of 2021 price data, which may not capture longer-term market fluctuations. Second, affordability estimates are based on national wage benchmarks, which mask regional variations. Third, distribution analysis is limited to state-level aggregates; more granular spatial data would allow advanced geospatial techniques such as Moran’s I or LISA cluster analysis to identify underserved hotspots. Finally, the study did not incorporate patient-level data or employ formal decomposition methods (e.g., Shapley value) to quantify the contribution of specific price drivers.

In sum, the findings reaffirm the potential of the Jan Aushadhi Scheme to reduce household expenditure on medicines, while also revealing persistent inequities and multidimensional barriers. Expanding equitable geographic coverage, addressing formulation-specific cost drivers, ensuring supply-chain reliability, and building public trust in generics through regulatory and educational measures will be critical to maximizing the scheme’s impact. Complementary research using richer datasets, spatial econometrics, and patient-centered evidence is essential to deepen understanding and strengthen the policy relevance of affordability studies in India.

## 5 Conclusion

This study provides new and critical insights into the affordability and accessibility of medicines under the Jan Aushadhi Scheme (JAS) in India. By systematically comparing JAS generics with branded alternatives across Eye and ENT medicines, and extending the analysis with illustrative chronic disease examples (Appendix Table A2) and dosage-form variations (Appendix Table A3), the findings demonstrate that the scheme offers substantial price relief but also reveal persistent inequities and structural barriers. While most JAS medicines fall within the WHO/HAI affordability threshold of 1 day’s wage, long-term treatments for chronic and ophthalmic conditions remain financially burdensome for poorer households, highlighting that affordability gains are uneven.

The expansion of Kendras between 2018 and 2022 underscores the strong policy commitment to scale-up; however, state-level and population-adjusted distribution metrics expose deep regional disparities that limit equitable access. Furthermore, regression and supplementary analyses confirm that competition alone does not ensure affordability, as price variation is strongly influenced by dosage form, packaging, patent exclusivity, and supply-chain inefficiencies. Counterfactual-style estimates indicate that scaling up generic substitution—for example, substituting branded Latanoprost with its JAS equivalent—could generate savings of several crores annually for every thousand patients, demonstrating the transformative financial protection potential of the scheme.

At the same time, barriers of perception and trust remain critical. Evidence from national surveys shows that rural households frequently cite cost and availability as barriers to healthcare access, and concerns about the quality of generics persist. Without addressing such behavioral and perceptual dimensions, the full benefits of JAS cannot be realized.

Taken together, the results highlight that expanding JAS is a necessary but insufficient condition for achieving medicine affordability in India. Stronger regulatory oversight of prescribing practices, equitable geographic rollout, improved supply-chain reliability, and public education to build confidence in generics are required to maximize the scheme’s impact. Future research should extend the therapeutic scope to chronic disease medicines, employ longitudinal and quasi-experimental designs to establish causal impacts, and incorporate patient-level perception and adherence data.

In conclusion, this study strengthens the evidence base on pharmaceutical affordability in India, while identifying actionable policy pathways to ensure that low-cost, high-quality medicines become equitably accessible to all populations, particularly the poorest and most vulnerable.

## Data Availability

This study analyzed publicly available datasets. The datasets used and analyzed during the current study are available from the corresponding author upon reasonable request.

## Footnotes

^1^Jan Aushadhi Kendras are government-supported retail outlets that sell generic medicines at affordable prices under the Jan Aushadhi Scheme.

## References

[1] Jamshidi-Kia F Lorigooini Z Amini-Khoei H. Medicinal plants: past history and future perspective. J Herbmed Pharmacol. (2017) 7:1–7. doi: 10.15171/jhp.2018.01, 39096352

[2] Yuan H Ma Q Ye L Piao G. The traditional medicine and modern medicine from natural products. Molecules. (2016) 21:559. doi: 10.3390/molecules21050559, 27136524 PMC6273146

[3] Yadav J Menon GR John D. Disease-specific out-of-pocket payments, catastrophic health expenditure and impoverishment effects in India: an analysis of National Health Survey Data. Appl Health Econ Health Policy. (2021) 19:769–82. doi: 10.1007/s40258-021-00641-9, 33615417

[4] Kankeu HT Saksena P Xu K Evans DB. The financial burden from non-communicable diseases in low-and middle-income countries: a literature review. Health Res Policy Syst. (2013) 11:1–12. 23947294 10.1186/1478-4505-11-31 PMC3751656

[5] Sharma K Secretary J Antony PJ Aushadhi Scheme J. For the use of members of parliament not for publication 1 Jan Aushadhi Scheme members’ reference service Larrdis Lok Sabha secretariat, New Delhi. Available online at: https://www.scribd.com/document/661697554/JAN-AUSHADHI-SCHEME

[6] Department of Pharmaceuticals. Annual Report (2020). Available online at: https://pharmaceuticals.gov.in/annual-report (accessed 30 November 2021)

[7] Dunne S Shannon B Dunne C Cullen W. A review of the differences and similarities between generic drugs and their originator counterparts, including economic benefits associated with usage of generic medicines, using Ireland as a case study. BMC Pharmacol Toxicol. (2013) 14:1–19. doi: 10.1186/2050-6511-14-1, 23289757 PMC3579676

[8] Alfonso-Cristancho R Andia T Barbosa T Watanabe JH. Definition and classification of generic drugs across the world. Appl Health Econ Health Policy. (2015) 13:5–11. doi: 10.1007/s40258-014-0146-1, 26091708 PMC4519628

[9] Wazana A. Physicians and the pharmaceutical industry: is a gift ever just a gift? JAMA. (2000) 283:373–80. doi: 10.1001/jama.283.3.373, 10647801

[10] Rataboli PV Garg A. Confusing brand names: nightmare of medical profession. J Postgrad Med. (2005) 51:1:13. 15793332

[11] Desai S Dass A Kaniganti S. Assessment of perception and attitude of postgraduates and clinicians toward generic versus branded medicines at a teaching medical institute. Natl J Physiol Pharm Pharmacol. (2018) 8:540–543. doi: 10.5455/njppp.2018.8.1144523112017

[12] Aivalli PK Elias MA Pati MK Bhanuprakash S Munegowda C Shroff ZC . Perceptions of the quality of generic medicines: implications for trust in public services within the local health system in Tumkur, India. BMJ Glob Health. (2017) 2:e000644. doi: 10.1136/BMJGH-2017-000644, 29531844 PMC5844374

[13] Toverud EL Hartmann K Håkonsen H. A systematic review of physicians’ and pharmacists’ perspectives on generic drug use: what are the global challenges? Appl Health Econ Health Policy. (2015) 13:35–45. doi: 10.1007/s40258-014-0145-2, 25963230 PMC4519583

[14] India Brand Equity Foundation. (2025). The Indian pharmaceutical industry. Available online at: https://www.ibef.org/industry/pharmaceutical-india

[15] World Health Organization. (2025). Global Health Observatory data repository. Available online at: https://www.who.int/data/gho (Accessed January 10, 2025).

[16] Cameron A Ewen M Auton M Abegunde D The world medicines situation 2011 medicines prices, availability and affordability. Geneva: World Health Organization. (2011).

[17] Mukherjee K. A cost analysis of the Jan Aushadhi scheme in India. Kerman Univ Med Sci. (2017) 6:253–6. doi: 10.15171/ijhpm.2017.02, 28812812 PMC5417146

[18] Yuvanesh P. Geetha P (2021). Cost comparison between branded medicines and Jan Aushadhi medicines. Annals of the Romanian Society for Cell Biology. 25:12345–12352. Available online at: https://www.annalsofrscb.ro/index.php/journal/article/view/8074/5946

[19] Bourne RRA Steinmetz JD Saylan M Mersha AM Weldemariam AH Wondmeneh TG . Causes of blindness and vision impairment in 2020 and trends over 30 years, and prevalence of avoidable blindness in relation to VISION 2020: the right to sight: an analysis for the global burden of disease study. Lancet Glob Health. (2021) 9:e144–60. doi: 10.1016/S2214-109X(20)30489-7, 33275949 PMC7820391

[20] Haile LM Kamenov K Briant PS Orji AU Steinmetz JD Abdoli A . Hearing loss prevalence and years lived with disability, 1990–2019: findings from the global burden of disease study 2019. Lancet. (2021) 397:996–1009. doi: 10.1016/S0140-6736(21)00516-X, 33714390 PMC7960691

[21] Ray A Najmi A Khandelwal G Sadasivam B. A cost variation analysis of drugs available in the Indian market for the management of thromboembolic disorders. Cureus. (2020) 12:e7964. doi: 10.7759/CUREUS.7964, 32523821 PMC7273361

[22] Atal S Mathur A Balakrishnan S. Cost of treating bacterial infections in India: a cost minimization analysis to assess price variations. Biomed Pharmacol J. (2020) 13:765–78. doi: 10.13005/bpj/1941

[23] Brennan H Kapczynski A Monahan CH Rizvi Z. A prescription for excessive drug pricing: leveraging government patent use for health. Yale JL & Tech. (2016) 18:275.

[24] Billa G Thakkar K Jaiswar S Dhodi D. A cross-sectional study to evaluate the awareness and attitudes of physicians towards reducing the cost of prescription drugs, Mumbai. Appl Health Econ Health Policy. (2014) 12:125–37. doi: 10.1007/s40258-014-0080-2, 24493092

[25] CIMS India. Search Drug Information, Interactions, Images, Dosage & Side Effects. Available online at: https://www.mims.com/india/ (Accessed January 10, 2025).

[26] Pharmaceuticals & Medical Devices Bureau of India. (PMBI). (2025). Available online at: http://janaushadhi.gov.in/index.aspx (Accessed January 10, 2025).

[27] Rao VR. Cost analysis study of Oral anti-diabetic drugs available in Indian govt generic drugs and brand drugs market in rural/ urban area of Guntur, India. World J Pharm Res. (2015)

[28] Bhaskarabhatla A Bhaskarabhatla A Mahagaonkar A. Regulating pharmaceutical prices in India. Cham, Switzerland: Springer (2018).

[29] Allan GM Lexchin J Wiebe N. Physician awareness of drug cost: a systematic review. PLoS Med. (2007) 4:1486–96. doi: 10.1371/journal.pmed.0040283, 17896856 PMC1989748

[30] Johnson N Somasundara Y Bhat P Kumar S Nayana M. Perception toward low-cost generic medicines and their usage among dental patients visiting community outreach programs in the peripheral areas of Bangalore south: an exploratory cross-sectional survey. J Indian Assoc Public Health Dent. (2020) 18:308. doi: 10.4103/jiaphd.jiaphd_80_20

[31] Roy V Rana P. Prescribing generics: all in a name. Indian J Med Res. (2018) 147:442–4. doi: 10.4103/ijmr.IJMR_1940_17, 30082567 PMC6094511

[32] Thawani V Mani A Upmanyu N. Why the Jan Aushadhi Scheme has lost its steam in India? J Pharmacol Pharmacother. (2017) 8:134–6. doi: 10.4103/jpp.JPP_38_17, 29081624 PMC5642129

[33] Smith A Johnson B Williams C. Current index of medical specialties (CIMS): drug pricing data. Bengaluru, India: CIMS Medical India Pvt. Ltd. (2021).

[34] Jones D. The importance of data quality in pharmaceutical research. J Med Econ. (2020) 15:123–35.

[35] Brown E Patel S. Analyzing drug pricing trends: a comprehensive guide. Pharm J. (2019) 25:67–78.

[36] Roberts L Kumar R. Accessing and utilizing drug pricing data: best practices for researchers. J Pharm Anal. (2020) 18:201–15.

[37] Gupta S. Role of CIMS in drug pricing: a review. Int J Pharm Sci. (2018) 12:45–58.

