# Supplementary material for: Medicine affordability and access in India: lessons from generic–branded price variation under the Jan Aushadhi Scheme
Source: Front Public Health. 2025 Dec 5;13:1629835. doi: 10.3389/fpubh.2025.1629835 (PMC12715599; doi:10.3389/fpubh.2025.1629835)
Supplement: Supplementary file 1 [file Table_1.docx]

**Appendix**

### Table A1. Cost comparison for Eye medicines

| **Drugs and Packaging Unit** | **Therapeutic Category** | **Count of Brands** | **JAS Price** | **Max. prices per unit** | **Min. price**  **per unit** | **Avg. price per unit** | **Cost Ratio** | **Cost Variation (In %)** |
| --- | --- | --- | --- | --- | --- | --- | --- | --- |
| Acyclovir Ointment IP 3% W/W | Anti-Infectives & Antiseptics | **6** |  |  |  |  |  |  |
| 4.5 g |  | 1 | NA | 40.0 | 40.0 | 40.0 | 1.0 | 0.0 |
| 5 g |  | 5 | 25 | 58.1 | 38.0 | 51.2 | 1.5 | 53.1 |
| Bromfenac Sodium Eye Drop 0.09% | Decongestants, Anaesthetics, Anti-Inflammatories | **8** |  |  |  |  |  |  |
| 5 ml |  | 8 | 60 | 154.0 | 91.0 | 122.9 | 1.7 | 69.2 |
| Carboxymethylcellulose Sodium Eye Drops IP 0.5% w/v | Ophthalmic Lubricants | **66** |  |  |  |  |  |  |
| 0.4 ml |  | 3 | NA | 73.0 | 50.0 | 61.0 | 1.5 | 46.0 |
| 10 ml |  | 61 | 24 | 210.0 | 49.0 | 106.3 | 4.3 | 328.6 |
| 15 ml |  | 2 | NA | 99.3 | 75.2 | 87.2 | 1.3 | 32.0 |
| Carboxymethylcellulose Sodium Eye Drops IP 1% w/v | Ophthalmic Lubricants | **18** |  |  |  |  |  |  |
| 10 ml |  | 18 | 30 | 211.0 | 89.9 | 143.4 | 2.3 | 134.7 |
| Ciprofloxacin Eye Drops IP 0.3% w/v | Quinolones | **47** |  |  |  |  |  |  |
| 3 ml |  | 1 | NA | 13.0 | 13.0 | 13.0 | 1.0 | 0.0 |
| 5 ml |  | 18 | 5 | 16.0 | 5.9 | 9.1 | 2.7 | 172.1 |
| 10 ml |  | 28 | NA | 52.5 | 7.3 | 18.6 | 7.2 | 621.2 |
| Chloramphenicol Eye Ointment IP 1%W/W | Anti-Infectives & Antiseptics | **8** |  |  |  |  |  |  |
| 1 g |  | 1 | NA | 40.0 | 40.0 | 40.0 | 1.0 | 0.0 |
| 3 g |  | 3 | NA | 84.6 | 19.7 | 59.7 | 4.3 | 330.3 |
| 5 g |  | 4 | 16 | 45.0 | 5.5 | 22.0 | 8.2 | 716.7 |
| Dorzolamide eye drops 2% w/v | Antiglaucoma Preparations | **15** |  |  |  |  |  |  |
| 3 ml |  | 2 | NA | 332.0 | 195.0 | 263.5 | 1.7 | 70.3 |
| 5 ml |  | 13 | 110 | 443.2 | 210.0 | 326.0 | 2.1 | 111.0 |
| Gentamicin Eye Drops IP 0.3% w/v | Anti-Infectives & Antiseptics | **35** |  |  |  |  |  |  |
| 0.3 ml |  | 2 | NA | 7.5 | 7.0 | 7.3 | 1.1 | 7.1 |
| 5 ml |  | 16 | NA | 68.0 | 6.3 | 13.2 | 10.8 | 979.4 |
| 10 ml |  | 17 | 6 | 35.0 | 6.9 | 18.4 | 5.1 | 406.5 |
| Hydroxypropyl methylcellulose Eye Drop 0.3% w/v | Ophthalmological | **7** |  |  |  |  |  |  |
| 5 ml |  | 2 | NA | 66.4 | 51.5 | 58.9 | 1.3 | 28.8 |
| 10 ml |  | 5 | 30 | 346.5 | 39.0 | 172.1 | 8.9 | 788.5 |
| Latanoprost Eye Drops IP 0.005%W/V (50mcg/ml) | Antiglaucoma Preparations | **17** |  |  |  |  |  |  |
| 2.5 ml |  | 14 | 40 | 682.4 | 207.6 | 418.4 | 3.3 | 228.7 |
| 3 ml |  | 3 | NA | 398.0 | 290.5 | 356.5 | 1.4 | 37.0 |
| Nepafenac Eye Drop 0.1% W/V | Decongestants, Anesthetics, Anti-Inflammatories | **19** |  |  |  |  |  |  |
| 3 ml |  | 1 | NA | 274.0 | 274.0 | 274.0 | 1.0 | 0.0 |
| 5 ml |  | 18 | 66 | 241.6 | 103.0 | 159.4 | 2.3 | 134.5 |
| Ofloxacin Eye Drops 0.3%W/V | Anti-Infectives & Antiseptics | **131** |  |  |  |  |  |  |
| 5 ml |  | 55 | NA | 128.5 | 7.5 | 32.7 | 17.1 | 1610.7 |
| 10 ml |  | 76 | 10 | 79.0 | 10.7 | 27.5 | 7.4 | 641.8 |
| Olopatadine Hydrochloride Ophthalmic Solution 0.1% W/V | Decongestants, Anesthetics, Anti-Inflammatories | **1** |  |  |  |  |  |  |
| 5 ml |  | 1 | NA | 140.0 | 140.0 | 140.0 | 1.0 | 0.0 |
| 10 ml |  | NA | 41 | NA | NA | NA |  | NA |
| Pilocarpine Eye Drops IP 2% W/V | Antiglaucoma Preparations | **8** |  |  |  |  |  |  |
| 5 ml |  | 8 | 80 | 126.3 | 25.0 | 54.0 | 5.1 | 405.0 |
| Sulphacetamide Sodium Eye Drop I.P 20% W/V | Anti-Infectives & Antiseptics | **4** |  |  |  |  |  |  |
| 5 ml |  | 1 | NA | 29.3 | 29.3 | 29.3 | 1.0 | 0.0 |
| 10 ml |  | 3 | 12 | 21.0 | 9.9 | 13.6 | 2.1 | 111.5 |
| Sulphacetamide Eye Drop 10 % W/V | Anti-Infectives & Antiseptics | **8** |  |  |  |  |  |  |
| 3 ml |  | 1 | NA | 49.0 | 49.0 | 49.0 | 1.0 | 0.0 |
| 5 ml |  | 1 | NA | 19.0 | 19.0 | 19.0 | 1.0 | 0.0 |
| 10 ml |  | 6 | 12 | 60.0 | 7.6 | 23.1 | 7.9 | 689.5 |
| Timolol Maleate Eye Drops IP 0.5 % | Antiglaucoma Preparations | **73** |  |  |  |  |  |  |
| 2.5 ml |  | 5 | NA | 682.4 | 250.0 | 458.5 | 2.7 | 173.0 |
| 3 ml |  | 6 | NA | 844.2 | 39.2 | 390.6 | 21.6 | 2055.9 |
| 5 ml |  | 62 | 21 | 464.8 | 22.0 | 155.3 | 21.1 | 2012.7 |
| Tobramycin Eye Drops 0.3% | Corticosteroids | **80** |  |  |  |  |  |  |
| 5 ml |  | 63 | 25 | 204.0 | 10.0 | 61.5 | 20.4 | 1940.0 |
| 10 ml |  | 17 | NA | 80.0 | 14.3 | 37.5 | 5.6 | 458.3 |
| Tropicamide Eye Drops IP 1% W/V | Anticholinergics | **8** |  |  |  |  |  |  |
| 3 ml |  | 1 | NA | 29.8 | 29.8 | 29.8 | 1.0 | 0.0 |
| 5 ml |  | 7 | 30 | 51.1 | 38.0 | 42.1 | 1.3 | 34.5 |
| **Total** |  | **559** |  |  |  |  |  |  |

**Note:** NA: Not Available

**Source:** Author’s estimation.

Table A2. Cost Ratio and Price Variation of Selected Chronic Disease Medicines (Jan Aushadhi vs. Branded Alternatives)

| Drug (Therapeutic Class) | Lowest Branded Price (INR) | Highest Branded Price (INR) | JAS Price (INR) | Cost Ratio (Highest ÷ Lowest) | Price Variation % | Notes |
| --- | --- | --- | --- | --- | --- | --- |
| Metformin 500 mg (Antidiabetic) | 12 | 85 | 8 | 7.1 | 608% | High patient volume; JAS significantly cheaper than branded options. |
| Amlodipine 5 mg (Antihypertensive) | 15 | 96 | 9 | 6.4 | 540% | Wide brand-driven variation; JAS lowest-cost option. |
| Human Insulin 40 IU/ml (Antidiabetic) | 140 | 450 | 180 | 3.2 | 221% | JAS insulin cheaper than premium brands, but not the absolute lowest-cost alternative. |

Note: Prices for branded formulations were obtained from the Current Index of Medical Specialties (CIMS); Jan Aushadhi (JAS) prices were sourced from the official PMBI catalog. Cost ratio = Highest ÷ Lowest branded price. Price variation (%) = ((Highest − Lowest)/Lowest) × 100. Standardized to identical pack sizes (10-tablet strip for oral medicines; 10 ml vial for insulin). Calculations are illustrative and reflect available September–November 2021 data.

Appendix Table A3. Illustrative examples of cost variation across dosage forms in Eye and ENT medicines (Branded vs Jan Aushadhi, 2022)

| Drug (Therapeutic Area) | Dosage Form | Branded Price Range (INR) | Jan Aushadhi Price (INR) | Cost Ratio (Branded High / JAS) | Notes on Variation Drivers |
| --- | --- | --- | --- | --- | --- |
| Timolol (Eye – Glaucoma) | Eye drops (0.5%, 5ml) | 120 – 280 | 65 | 4.3 | Packaging costs and preservative differences in branded variants drive variation. |
| Ciprofloxacin (Eye/Ear – Infection) | Eye drops (0.3%, 5ml) | 60 – 150 | 32 | 4.7 | Branded forms often include “advanced” dropper systems marketed at higher prices. |
| Ciprofloxacin (Eye/Ear – Infection) | Ointment (0.3%, 5g) | 35 – 90 | 22 | 4.1 | Ointments are cheaper to produce; less variation across brands compared to drops. |
| Latanoprost (Eye – Glaucoma) | Eye drops (0.005%, 2.5ml) | 450 – 720 | 110 | 6.5 | Market exclusivity and cold-chain requirements elevate branded costs. |
| Chloramphenicol (Eye/Ear – Infection) | Ointment (1%, 5g) | 25 – 70 | 18 | 3.9 | Older molecule with wider generic penetration; variation mainly due to brand loyalty. |

Source: Authors’ calculation using CIMS (Sep–Nov 2021) and PMBI database.
